# Data supporting the assessment of biomass based electricity and reduced GHG emissions in Cuba

**DOI:** 10.1016/j.dib.2018.01.071

**Published:** 2018-02-01

**Authors:** Alexis Sagastume Gutiérrez, Juan J. Cabello Eras, Carlo Vandecasteele, Luc Hens

**Affiliations:** a Universidad de la Costa, Calle 50 No 55-66, PBX 336 22 00 Barranquilla, Colombia; b Universidad de Cienfuegos, Carretera a Rodas kilómetro 4, Cuatro caminos, Cienfuegos, Cuba; c Department of Chemical Engineering, KU Leuven, Celestijnenlaan 200F, 3001 Leuven, Belgium; d Vlaamse Instelling voor Technologisch Onderzoek (VITO), Boeretang 200, B2400 Mol, Belgium

## Abstract

Assessing the biomass based electricity potential of developing nations like Cuba can help to reduce the fossil fuels dependency and the greenhouse gas emissions. The data included in this study present the evolution of electricity production and greenhouse gas emissions in Cuba. Additionally, the potentialities to produce biomass based electricity by using the most significant biomass sources in Cuba are estimated. Furthermore, estimations of the potential reductions of greenhouse gas emissions, resulting from implementing the biomass based electricity potential of the different sources discussed in the study, are included. Results point to the most promising biomass sources for electricity generation and their potential to reduce GHG emissions.

## Specifications table

**Table:** 

Subject area	*Renewable energy, environment*
More specific subject area	*Carbon dioxide emissions.*
Type of data	*Table*
How data was acquired	*From documents and own calculations.*
Data format	*Raw, filtered, analyzed, etc.*
Data source location	*Cuba*
Data accessibility	*Data is available at*www.one.cu
	*Complementary data is available in literature (see reference list)*
Related research article	*The current potential of low-carbon economy and biomass-based electricity in Cuba. The case of sugarcane, energy cane and marabu (dichrostachys cinerea) as biomass sources “in press”.*

## Value of the data

• This data contains key information for the biomass production and the GHG emissions in Cuba.
• This data can be used to estimate the biomass based electricity potential of Cuba.
• This data can be used to estimate the reduction of GHG emissions that could result from implementing the different biomass based electricity potentialities existing in Cuba.
• This data permits to focus on the largest biomass sources for energy production in Cuba.

## 1 Data

The data presented in the article is related to the research article: *The current potential of low-carbon economy and biomass-based electricity in Cuba. The case of sugarcane, energy cane and marabu (dichrostachys cinerea) as biomass sources* [1]. The data corresponds to the evolution of the electricity production and of the GHG emissions in Cuba, and includes the biomass potential of the largest sources and the estimation of the associated biomass based electricity generation and greenhouse gas (GHG) emissions potential. The data of the evolution of the electricity production and of the GHG emissions was collected from the National Statistics Office of Cuba, when needed complemented with information from literature and databases. The estimations of the potentialities of biomass based electricity production and GHG emissions reduction in Cuba are calculated to highlight the main features.

## 2 Materials and methods

Based on the available biomass sources (between 2011 and 2016) estimations of the biomass based electricity potential and the possibilities to reduce GHG are developed. The biomass based electricity potential was calculated as:

(1) $$E=\mathrm{LH}V_{w}\cdot \eta_{\mathrm{elect}}$$

where:

*E* – Electricity potential (kWh/t)
$\eta_{\mathrm{elect}}$ – Electricity efficiency of the generation technology (understand as the % of the *LHV*_W.B._ transformed into electricity)

An electricity production efficiency of 28% was considered for biomass incineration [1,3]. Moreover, to assess the potentialities of pig manure, where the use of the biogas resulting from manure rather than directly incinerating manure (because of its high moisture content) is considered, an electricity production efficiency of 35% was used [2].

The factors used to estimate the biomass resulting from the production of different crops and livestock in Cuba are included in Table 1. For the estimations of dichrostachys cinerea (kwon as marabu, is a non-indigenous bush tree that is widely available and considered a fast spreading plague, occupying between 1.5 and 2 million ha) it is considered that between 2011 and 2016 its area increased from 1.5 to 2 million ha (at a rate of 100,000 ha/year). Marabu yields 37 t/ha with a re-grow period of three years [3]. Based on re-grow period, the yearly marabu based electricity potential is estimated as 33.3% of the overall potential of the marabu stock.

**Table 1:** Biomass production factors.

**Biomass**	**Sugarcane (t)**	**Paddy rice (t)**	**Poultry (head)**	**Pig (head)**	**Pig manure (t)**	**Ref.**
Filter cake (kg)	33	–	–	–	–	[5]
Rice husk (t)	–	0.22	–	–	–	[6]
Drying wastes (t)	–	0.04	–	–	–	[6]
Poultry manure kg)	–	–	0.12	–	–	[7]
Pig manure (kg)	–	–	–	794.7	–	[8]
Biogas (m^3^)	–	–	–	–	14	[9]

**Fig. 1:**
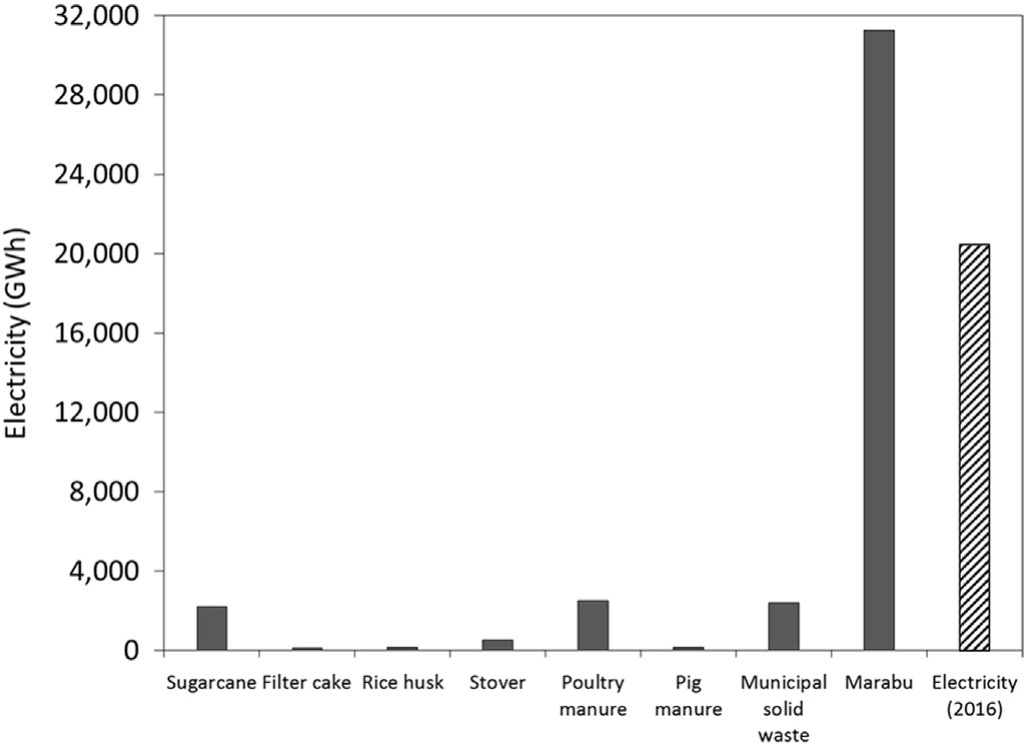
Biomass based electricity potential of the biomass sources vs electricity generation in Cuba (2016).

**Fig. 2:**
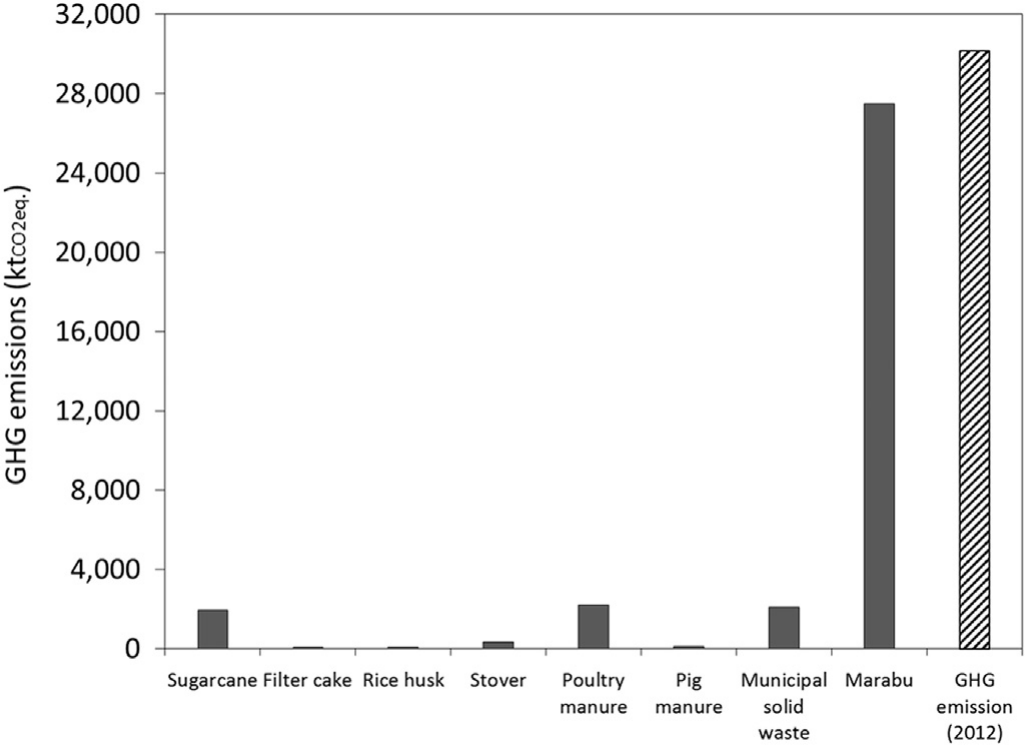
Potential of GHG emission reductions of the biomass sources vs GHG emissions in Cuba (2012).

To assess the potential reductions of the GHG emissions, it is considered that the GHG emissions of producing the different crops and livestock are allocated to the production of the product (e.g. rice, maize grain, meat, eggs, sugar, etc.). This is not entirely true since biomass is not carbon neutral. However, it serves as a first approximation. Thus, it is considered that biomass based electricity can save 100% of the GHG emissions resulting from generating the same amount of fossil based electricity. In Cuba, the greenhouse gas emission factor for electricity generation is 0.879 t_CO2_eq./MWh [4] (Figs. 1 and 2, Table 2, Table 3, Table 4, Table 5, Table 6, Table 7, Table 8, Table 9).

**Table 2:** Evolution of power generation and GHG emissions in Cuba.

**Year**	**Power (MW)**	**Electricity (GWh)**	**FBE (GWh)**	**SBE (GWh)**	**HP (GWh)**	**GHG.W (kt**_**CO2eq.**_**)**	**Year**	**Power (MW)**	**Electricity (GWh)**	**FBE (GWh)**	**SBE (GWh)**	**HP (GWh)**	**ESPE (GWh)**	**GHG.C (kt**_**CO2eq.**_**)**	**GHG.W (kt**_**CO2eq.**_**)**
**1959**	475.6	2348.4	1956.4	392.0	5.9	–	**1988**	3841.3	14,542.3	13,225.0	1317.3	72.8	0.0	–	35,636
**1960**	472.6	2492.7	2105.7	387.0	13.0	13,700	**1989**	3998.9	15,239.8	13,959.7	1280.1	82.0	0.0	–	35,739
**1961**	509.1	2521.9	2086.9	435.0	8.5	12,182	**1990**	4077.9	15,024.7	13,575.6	1449.1	90.9	0.0	15,025	33,344
**1962**	534.1	2552.5	2257.5	295.0	8.6	14,169	**1991**	4033.3	13,247.2	11,982.8	1264.4	104.7	0.0	–	29,710
**1963**	532.1	2597.0	2345.0	252.0	49.8	13,040	**1992**	4032.2	11,538.0	10,200.8	1337.2	80.5	0.0	22,934	31,294
**1964**	566.1	2811.4	2494.6	316.8	100.5	14,294	**1993**	4031.7	11,004.2	10,117.0	887.2	82.4	0.0	–	29,380
**1965**	564.1	2954.5	2954.5	–	56.7	14,609	**1994**	4059.6	11,964.0	11,067.1	896.9	48.5	0.0	23,192	32,248
**1966**	658.6	3157.4	3157.4	–	131.4	15,185	**1995**	3991.1	12,459.0	11,769.3	689.7	74.4	0.0	–	25,709
**1967**	758.6	3453.6	3453.6	–	109.2	15,750	**1996**	4311.9	13,236.5	12,314.4	922.1	95.2	0.0	27,284	26,996
**1968**	861.5	3615.4	3615.4	–	80.7	16,036	**1997**	4223.9	14,145.6	13,275.9	869.7	130.0	0.0	–	24,650
**1969**	913.5	3782.3	3782.3	–	102.9	17,261	**1998**	4348.3	14,148.6	13,369.5	779.1	96.7	0.0	28,886	24,499
**1970**	908.0	4888.5	4008.0	880.5	90.7	18,672	**1999**	4284.3	14,492.2	13,611.3	880.9	103.3	0.0	–	25,332
**1971**	985.0	5020.5	4203.5	817.0	110.2	19,607	**2000**	4286.5	15,032.2	14,088.0	944.2	89.0	0.0	27,558	26,083
**1972**	1466.2	5269.0	4624.0	645.0	74.0	20,799	**2001**	4410.9	15,299.8	14,369.5	930.3	75.0	0.0	–	25,453
**1973**	1531.8	5707.9	4989.0	718.9	62.0	22,398	**2002**	3959.6	15,698.8	14,760.3	938.5	106.4	0.3	25,786	26,091
**1974**	1644.6	6019.6	5283.4	736.2	89.4	22,911	**2003**	3965.0	15,810.5	15,090.4	720.1	127.7	0.4	–	25,486
**1975**	1677.3	6588.9	5831.8	756.2	62.5	27,066	**2004**	3763.5	15,633.7	14,845.1	788.6	87.6	0.4	25,266	25,005
**1976**	1704.6	7195.9	6422.6	773.3	53.2	27,224	**2005**	4275.1	15,341.1	14,921.6	419.5	67.7	0.1	–	26,006
**1977**	1858.0	7705.0	6868.9	836.1	72.8	29,402	**2006**	5176.0	16,468.5	16,062.4	406.1	93.5	0.3	28,829	27,407
**1978**	2288.3	8482.7	7527.0	955.7	83.2	30,689	**2007**	5429.4	17,622.5	17,209.7	412.8	121.4	0.2	–	26,795
**1979**	2560.7	9403.1	8445.0	958.1	104.3	31,712	**2008**	5396.4	17,661.8	17,127.6	553.7	138.3	8.2	32,216	30,443
**1980**	2731.4	9989.6	9035.4	954.2	97.1	31,401	**2009**	5550.0	17,727.1	17,037.9	534.8	150.8	3.6	–	29,897
**1981**	2751.8	10,575.5	9600.1	975.4	59.8	32,750	**2010**	5852.6	17,395.5	16,832.3	446.2	96.6	11.7	30,378	38,375
**1982**	2974.5	11,071.4	10,025.9	1045.5	42.7	34,554	**2011**	5913.9	17,754.1	17,186.6	453.8	99.2	19.8	–	35,988
**1983**	2999.9	11,551.4	10,466.6	1084.8	62.7	30,843	**2012**	5699.1	18,427.9	17,744.3	551.0	110.9	21.7	30,173	36,157
**1984**	3111.2	12,292.0	11,167.3	1124.7	70.4	32,603	**2013**	6054.8	19,139.6	18,306.9	696.6	127.3	25.6	–	34,800
**1985**	3249.0	12,199.4	11,068.0	1131.4	54.3	32,578	**2014**	6168.6	19,366.1	18,588.3	636.5	104.1	37.2	–	34,837
**1986**	3419.2	13,176.4	11,991.7	1184.7	59.3	33,568	**2015**	6280.0	20,288.0	19,585.3	702.7	48.3	50.1	–	–
**1987**	3532.0	13,594.0	12,388.8	1204.7	43.9	33,953	**2016**	6453.9	20,458.6	19,648.0	686.3	64.2	–	–	–

* FBE – Fossil based electricity, SBE – Sugarcane based electricity, HE – Hydroelectricity, ESPE – Eolic + Solar photovoltaic, GHG.C – Net GHG emissions reported by the Cuban government, GHG.W – Net GHG emissions reported by the World Bank.

**Table 3:** Evolution of sugarcane production and its use of agricultural land in Cuba.

**Year**	**Harvested surface (ha)**	**Yield (t)**	**Production (t)**	**Bagasse (t)**	**Year**	**Harvested surface (ha)**	**Yield (t)**	**Production (t)**	**Bagasse (t)**
**1959**	1,070,000	41.9	44,800,000	12,960,000	**1988**	1,297,300	56.8	76,714,080	21,819,600
**1960**	1,160,000	40.9	47,500,000	12,203,300	**1989**	1,350,600	60.0	85,218,000	23,022,700
**1961**	1,260,000	43.1	54,300,000	14,002,700	**1990**	1,420,300	57.6	83,646,720	23,261,900
**1962**	1,130,000	32.5	36,700,000	9,724,600	**1991**	1,452,200	54.9	79,698,330	19,473,800
**1963**	1,070,000	29.3	31,400,000	8,386,100	**1992**	1,451,700	45.6	55,253,520	10,093,300
**1964**	1,000,000	37.2	37,200,000	9,880,200	**1993**	1,211,700	36.0	44,960,400	12,921,200
**1965**	1,060,000	47.8	50,700,000	13,344,100	**1994**	1,248,900	34.6	40,738,040	12,902,700
**1966**	980,000	37.0	36,800,000	9,874,900	**1995**	1,177,400	28.5	35,468,250	10,208,100
**1967**	1,040,000	35.0	50,500,000	13,950,300	**1996**	1,244,500	33.2	41,377,160	12,423,200
**1968**	1,010,000	42.4	42,800,000	11,869,000	**1997**	1,246,300	31.2	32,713,200	11,859,500
**1969**	940,000	44.4	41,700,000	11,551,400	**1998**	1,048,500	31.3	31,168,540	10,070,300
**1970**	1,460,000	55.8	81,500,000	23,274,100	**1999**	995,800	34.1	35,494,690	10,673,300
**1971**	1,250,000	41.7	52,200,000	15,836,700	**2000**	1,040,900	35.6	35,852,760	11,038,700
**1972**	1,180,000	37.5	44,300,000	13,369,100	**2001**	1,007,100	31.4	32,693,680	11,599,000
**1973**	1,070,000	45.0	48,200,000	14,254,000	**2002**	1,041,200	33.3	21,438,540	8,952,000
**1974**	1,100,000	45.8	50,400,000	14,779,200	**2003**	643,800	34.3	22,672,300	7,100,700
**1975**	1,180,000	44.4	52,400,000	15,153,300	**2004**	661,000	36.0	18,619,200	6,950,500
**1976**	1,220,000	44.1	53,800,000	15,275,800	**2005**	517,200	22.4	8,895,040	4,787,300
**1977**	1,140,000	53.0	60,400,000	16,073,200	**2006**	397,100	28.0	9,226,000	3,605,800
**1978**	1,240,000	56.1	69,600,000	18,678,800	**2007**	329,500	36.1	13,728,830	3,415,100
**1979**	1,310,000	59.0	77,300,000	19,585,100	**2008**	380,300	41.3	17,953,110	3,863,300
**1980**	1,390,000	46.0	64,000,000	17,108,000	**2009**	434,700	34.3	14,797,020	3,719,000
**1981**	1,210,000	55.0	66,600,000	19,147,000	**2010**	431,400	26.7	13,512,870	3,027,300
**1982**	1,330,000	55.0	73,100,000	19,075,000	**2011**	506,100	31.2	11,272,560	3,949,600
**1983**	1,200,000	58.1	67,400,000	19,149,000	**2012**	361,300	39.9	15,971,970	3,959,900
**1984**	1,350,000	57.3	77,400,000	19,635,000	**2013**	400,300	40.3	16,329,560	3,637,100
**1985**	1,347,800	50.0	67,400,000	18,315,000	**2014**	405,200	44.10	19,300,000	4,604,200
**1986**	1,328,600	51.6	70,088,280	19,584,000	**2015**	435,600	44.30	19,297,080	4,942,000
**1987**	1,358,300	52.1	67,589,330	19,969,000	**2016**	–	–	15,806,667	3,793,600

**Table 4:** Biomass properties and electric potential.

**Biomass**	**Moisture (%)**	**HHV**_**d**_**(MJ/kg)**	**LHV**_**w**_**(MJ/kg)**	**Electricity potential (kWh/t)**	**Ref.**
**Bagasse**	50.0%	17.30	7.43	577.6	[3]
**Filter cake**	40.0%	14.50	7.72	600.5	[3]
**Marabu**	19.0%	20.70	16.30	1267.9	[3]
**Rice husk**	9.0%	16.50	14.79	1150.7	[14]
**Maize**	6.1%	–	15.68	1219.6	[15]
**Poultry manure**	39.7%	–	8.54	664.2	[15]
**Pig manure**	92.1%	–	− 1.24	0	[15]
**Biogas from pig manure**	–	–	18	51.7	[9]
**Municipal solid waste**	44.0%	–	7.15	556	[16]

**Table 5:** Production of the main crops, livestock and municipal solid wastes in Cuba: 2011–2016.

**Year**	**Sugarcane (t)**	**Maize (t)**	**Paddy rice (t)**	**Poultry (heads)**	**Pig (heads)**	**Municipal solid waste (m**^**3**^**)**
**2011**	11,272,560	304,800	3,256,100	33,663,300	3,256,100	23,390,400
**2012**	15,971,970	324,463	3,036,100	30,182,000	3,036,100	27,817,400
**2013**	16,329,560	354,000	3,366,700	32,415,500	3,366,700	26,521,000
**2014**	19,300,000	360,400	3,379,600	32,285,800	3,379,600	27,221,300
**2015**	19,297,080	426,200	3,492,800	31,963,900	3,492,800	28,007,800
**2016**	15,806,667	427,295	3,600,800	31,336,200	3,600,800	28,796,400

**Table 6:** Estimation of the biomass production from the more significant sources in Cuba: 2011–2016.

**Year**	**Bagasse (t)**	**Filter cake (t)**	**Rice husk (t)**^a^	**Stover (t)**	**Poultry manure (t)**	**Pig manure (t)**	**Municipal solid waste (t)**^b^	**Total (t)**
**2011**	3,949,600	371,994	147,264	304,800	4,039,596	2,587,623	3,508,560	14,909,437
**2012**	3,959,900	527,075	166,816	324,463	3,621,840	2,412,789	4,172,610	15,185,493
**2013**	3,637,100	538,875	174,876	354,000	3,889,860	2,675,516	3,978,150	15,248,378
**2014**	4,604,200	636,900	152,048	360,400	3,874,296	2,685,768	4,083,195	16,396,807
**2015**	4,942,000	636,804	108,690	426,200	3,835,668	2,775,728	4,201,170	16,926,259
**2016**	3,793,600	521,620	133,652	427,295	3,760,344	2,861,556	4,319,460	15,817,526

a Includes rice husk and drying wastes.

b A density of 150 kg/m^3^ is considered for Municipal Solid Waste.

**Table 7:** Estimation of the marabu (dichrostachys cinerea) biomass stock in Cuba: 2011–2016.

**Year**	**Surface (ha)**	**Biomass (t)**
**2011**	1,500,000	55,500,000
**2012**	1,600,000	59,200,000
**2013**	1,700,000	62,900,000
**2014**	1,800,000	66,600,000
**2015**	1,900,000	70,300,000
**2016**	2,000,000	74,000,000

**Table 8:** Calculation of biomass based electricity potential in Cuba: 2011–2016.

**Year**	**Sugarcane (GWh)**	**Rice husk (GWh)**	**Stover (GWh)**	**Poultry manure (GWh)**	**Pig manure (GWh)**	**Municipal solid waste (GWh)**	**Marabu (GWh)**	**Total (GWh)**
**2011**	1578	169	372	2683	134	1951	70,371	77,336
**2012**	2236	192	396	2406	125	2320	75,062	82,848
**2013**	2286	201	432	2584	138	2212	79,753	87,720
**2014**	2702	175	440	2573	139	2271	84,445	92,878
**2015**	2702	125	520	2548	144	2336	89,136	97,644
**2016**	2213	154	521	2498	148	2402	93,827	101,873

**Table 9:** Calculation of the biomass based GHG reduction potential in Cuba: 2011–2016.

**Year**	**Sugarcane (kt**_**CO2.eq**_**)**	**Rice husk (kt**_**CO2.eq**_**)**	**Stover (kt**_**CO2.eq**_**)**	**Poultry manure (kt**_**CO2.eq**_**)**	**Pig manure (kt**_**CO2.eq**_**)**	**Municipal solid waste (kt**_**CO2.eq**_**)**	**Marabu (kt**_**CO2.eq**_**)**	**Total (kt**_**CO2.eq**_**)**
**2011**	1387	112	245	2359	118	1715	61,856	67,860
**2012**	1966	126	261	2115	110	2040	65,979	72,694
**2013**	2010	133	285	2271	122	1945	70,103	76,967
**2014**	2375	115	290	2262	122	1996	74,227	81,505
**2015**	2375	82	343	2239	126	2054	78,351	85,687
**2016**	1945	101	343	2195	130	2111	82,474	89,398
