# Supplementary material for: Data supporting the assessment of biomass based electricity and reduced GHG emissions in Cuba
Source: Data Brief. 2018 Feb 1;17:716–23. doi: 10.1016/j.dib.2018.01.071 (PMC5832642; doi:10.1016/j.dib.2018.01.071)
Supplement: Supplementary file 1 — Supplementary material [file mmc1.docx]

Conflict of interest form:

We have no conflict of interest to declare.
